# Assessment of Renal Function of Bodybuilders Using Anabolic Androgenic Steroids and Diet Supplements

**DOI:** 10.7759/cureus.43058

**Published:** 2023-08-07

**Authors:** Sultan Ozkurt, Engin Ozakin, Hilal Gungor, Ahmet Ugur Yalcin

**Affiliations:** 1 Nephrology, Faculty of Medicine, Eskişehir Osmangazi Üniversitesi, Eskişehir, TUR; 2 Emergency Medicine, Faculty of Medicine, Eskişehir Osmangazi Üniversitesi, Eskişehir, TUR; 3 Internal Medicine, Eskişehir City Hospital, Eskişehir, TUR

**Keywords:** glomerular filtration rate, renal side effects, diet supplements, bodybuilders, anabolic androgenic steroids

## Abstract

Introduction

Anabolic androgenic steroids (AAS) and diet supplements (DS) are frequently used by bodybuilders. In this specific group, increased muscle mass, the acute effects of exercise, and the use of creatine may affect the creatinine-based estimated glomerular filtration rate (eGFRcr), potentially leading to an underestimation of the GFR. Cystatin C equations offer a more accurate prediction of GFR that is independent of muscle mass. We aimed to assess the renal functions of bodybuilders who use both AAS+DS, as well as those who only use DS, by calculating the GFR based on cystatin C (eGFRcys) and also using a combination of cystatin C and creatinine (eGFRcys/cr).

Methods

The study included 12 bodybuilders using AAS+DS and 12 bodybuilders using DS. In both groups, serum cystatin C levels, eGFRcys, eGFRcys/cr, urine albumin excretion rates, urine protein excretion rates, and routine tests were examined.

Results

In AAS+DS users, the average duration of AAS use was 3.08±2.02 years, while for DS users, the duration of supplement use was 3.67±2.49 years. The spot urine albumin/creatinine and protein/creatinine ratios were higher in AAS+DS users (p<0.001 and p=0.006, respectively). Although eGFRcr was found to be similar in the AAS+DS and DS groups (119.67 ± 24.12 ml/min and 122.08 ± 18.03 ml/min, respectively; p=0.426), eGFRcys and the eGFRcys/cr ratio were significantly lower in the AAS+DS group compared to the DS group (eGFRcys: 120.67 ± 19.48 ml/min vs. 122.08 ± 18.03 ml/min, p=0.039; eGFRcys/cr: 121.83 ± 20.62 ml/min vs. 126.33 ± 21.163 ml/min, p= 0.036, respectively).

Conclusion

Cystatin-based GFR values were found to be significantly lower in AAS+DS users, and urinary albumin and protein excretion were considerably higher compared to DS users. Although these findings suggest a potential link between early kidney damage and the direct use of AAS, the topic requires further investigation.

## Introduction

Performance-enhancing substances (PES) are commonly used by sportspeople to improve athletic performance or physical appearance. One of the best-known of these substances is anabolic androgenic steroids (AAS) [1]. These drugs have been used in the last five decades to increase muscle mass in bodybuilders and people who want masculine physiques and to intensify exercise regimes [2]. In recent years, AAS consumption in Western countries has been increasing among young people [3, 4]. In addition to AAS use, some diet supplements (DS) sold in the market are proposed to improve athletic/physical performance. The most frequently used non-hormone supplements are protein powder, vitamins, and creatine. Despite some of these substances being used as supplements to replenish nutrients lost during physical activity, others mimic the effects of AAS and may have similar associated effects [1]. The continuous use of AAS for therapeutic purposes may involve some side effects and health problems. The known general side effects of AAS include hypertension, accelerated atherosclerosis, reduced fertility, liver toxicity, and immune system suppression [5]. Athletes use these drugs in 8-12 week cycles, and these general side effects are not well-documented in athletes. The renal side effects of these drugs are less known. The renal side effects of these drugs became a topic of curiosity after Herlitz LC et al. showed the association of focal segmental glomerulosclerosis (FSGS) and proteinuria after long-term use of AAS in a cohort composed of 10 bodybuilders [6]. A high-protein diet (300-550 g/day) is known to increase the glomerular filtration rate (GFR) and has been experimentally reported to cause glomerular hyperfiltration and FSGS [7]. Creatine powders are believed to be effective in increasing exercise tolerance and muscle power and are considered safe for use at 20-25 g/day for a 5-day loading phase, followed by a 5 g/day maintenance dose [8, 9]. There are few reports related to the side effects of creatine powders; these are generally about acute renal injury developing due to rhabdomyolysis linked to intense exercise [10]. Endogenous biomarkers are widely used to estimate GFR. Serum creatinine continues to be the most commonly used biomarker for this purpose. Recently, the combination of cystatin C and creatinine has been shown to be the best approach for calculating GFR based on endogenous biomarkers [11, 12]. In bodybuilders, high muscle mass may cause serum creatinine values to be measured higher than they actually are, leading to falsely elevated serum creatinine levels and, consequently, falsely low GFR values. Equations using estimated GFR (eGFR) measured with creatinine have proven validity for most patients, but their accuracy has not been confirmed for those with extremely muscular body composition. Additionally, since many of these patients take creatine supplements, and creatine is easily converted to creatinine, using creatinine measurements to estimate GFR in this population may create a potential problem [13]. Cystatin C, produced at a fixed rate by all nucleated cells, has no clear diurnal rhythm within the day. Its fixed production rate, free release from glomeruli, and lack of influence due to body muscle mass make it a more sensitive parameter for assessing GFR than creatinine. Additionally, cystatin C serum levels do not display variation linked to age and sex [14, 15].

In this study, we aimed to assess the renal functions of bodybuilders using AAS+DS and DS alone by calculating GFR based on cystatin C and the combination of cystatin C and creatinine.

## Materials and methods

Participants

The study included 24 male volunteers aged 25-35 years (12 bodybuilders using AAS+DS and 12 bodybuilders using DS) attending gyms in Eskişehir. The mean age of AAS+DS users was 32.33 ± 5.12 years, BMI was 27.07 ± 2.53 (kg/m²), the mean age of DS users was 29.50 ± 4.77 years, and BMI was 25.65 ± 1.76 (kg/m²). Those with diabetes mellitus, hypertension, and chronic kidney disease history were excluded from the study. The study was permitted by Eskişehir Osmangazi University Clinical Research Ethics Committee on May 20, 2019, with decision number 22. The Republic of Türkiye Ministry of Health, Turkey Pharmaceuticals and Medical Devices Agency granted permission on August 1, 2019, dated 66 175679-5 14.99-E.120952. All participants gave informed consent.

Questionnaire and data collection

Volunteers were asked about the types, amounts, and duration of AAS and DS they used, along with stimulants (like amphetamines and cocaine), smoking and alcohol habits, sociodemographic characteristics, sports and physical activity, and any side effects of the agents used.

Additionally, participants had their blood pressure and heart rate measured after a minimum of 5 minutes of rest.

Renal ultrasound and dimensions of both kidneys were examined by a lecturer certified in the use of ultrasound. Imaging was done using a 3.5-5 MHz convex probe with a Samsung HS50 Hybrid ultrasound machine. The kidneys were examined in both longitudinal and transverse scan planes, with the transducer placed in the flanks.

Sample analysis

Blood and urine samples taken for routine tests were analyzed on the same day. Full blood count was examined with a Sysmex XN-1000 using full blood samples in K2EDTA. Blood samples for routine biochemistry tests were centrifuged for 10 minutes at 1500 g and after separation were studied with a Cobas 702 modular autoanalyzer (Roche Diagnostics, Mannheim, Germany) with the colorimetric method using available commercial kits. Serum samples had TSH and total testosterone examined with a Cobas 801 autoanalyzer (Roche Diagnostics, Mannheim, Germany) with the electrochemiluminescence immune method (ECLIA). Full urine tests were studied with microscopy and chemical strip analyses using an FUS 200 urine analyzer (Iris Diagnostics, Chatsworth, CA, USA). For cystatin C, blood samples were centrifuged for 10 minutes at 3000 g and serum samples were stored at -80 degrees until analysis. Total protein in urine samples was analyzed using the turbidimetric method, while albumin was examined with the immunoturbidimetric method. Creatinine tests were conducted using the kinetic colorimetric Jaffé method with a Cobas 501 autoanalyzer (Roche Diagnostics, Mannheim, Germany).

Measurement of human serum cystatin C levels

In serum, cystatin C measurements were performed with a BioVendor brand Human Cystatin C Elisa Kit brand Human Cystatin C ELISA measurement kit (BioVendor R&D, Cystatin C ELISA, RD191009100). Absorbance was read with a Chromate 4300 brand ELISA reader device (Awareness Technology, Inc. Martin Hwy., USA). Results are given as mg/L.

Statistics

Continuous data are presented as the mean ± SD, and categorical data are given as percentages (%). The fit of the data to a normal distribution was tested with the Shapiro-Wilk test. Comparison of groups with normal distribution used the independent samples t-test analysis for situations with groups numbering two. Comparison of groups without normal distribution used the Mann-Whitney U test for groups of two. Analysis of the cross tables used the Pearson chi-square and Pearson exact chi-square analyses. To implement the analyses, the IBM SPSS Statistics 21.0 (IBM Corp., Armonk, NY, USA) program was used. For statistical significance, p<0.05 was accepted as the criterion.

## Results

For both groups, age, BMI, educational level, systolic blood pressure (SBP), diastolic blood pressure (DBP), heart rate, and weekly meat consumption amounts were similar, as summarized in Table 1.

**Table 1:** Physical examination findings and demographic characteristics of the participants. * Independent sample t-test **Mann-Whitney U test *** Fisher exact Chi-square test AAS: Anabolic androgenic steroid; DS: Dietary supplement.

	AAS+DS	DS	P-value
Age (years)	32.33 ± 5.12	29.50 ± 4.77	0.184 *
BMI (kg/m²)	27.07 ± 2.53	25.65 ± 1.76	0.207 *
Education level (high school/university)	2/10 (16.7%/83.3%)	1/11 (8.3%/91.7%)	1.00 ***
Systolic blood pressure (mm/Hg)	122.10 ± 15.03	117.18 ± 22.96	0.194 *
Diastolic blood pressure (mm/Hg)	71.20 ± 7.95	69.55 ± 12.69	0.104 *
Heart rate (beats/min)	72.70 ± 11.22	78.64 ± 12.10	0.889 *
Meat consumption (kg/wk)	3500.0 ± 1804.3	2000.00 ± 1723.6	0.289 **
Right kidney length (cm)	10.60 ± 0.98	10.20 ± 1.22	0.128 *
Left kidney length (cm)	10.71 ± 1.12	9.78 ± 0.83	0.617 *

Those using AAS+DS used AAS for 3.08±2.02 years, while DS users used them for 3.67±2.49 years (p=0.906). Table 2 summarizes the types of AAS used, weekly AAS amounts, and durations for patients using AAS.

**Table 2:** AAS use by twelve bodybuilders. AAS: Anabolic androgenic steroid.

	AAS Type	AAS amount/week	Total ASS amount/week	Usage time (years)	Frequency of use
Patient 1	Testosterone propionate Drostanolone propionate Stanozolol Oxandrolone	500 mg 300 mg 50 mg 50 mg	900 mg	4 y	4 months every 2 years
Patient 2	Testosterone propionate Drostanolone propionate Trenbolone acetate	500 mg 200 mg 200 mg	900 mg	5 y	2 months a year
Patient 3	Testosterone propionate Drostanolone propionate Boldenone	500 mg 200 mg 500 mg	1200 mg	6 y	4 months a year
Patient 4	Testosterone propionate Drostanolone propionate Stanozolol	200 mg 200 mg 100 mg	500 mg	1 y	3 months a year
Patient 5	Testosterone propionate	200 mg	200 mg	4 y	1 month a year
Patient 6	Testosterone propionate Drostanolone propionate	200 mg 200 mg	400 mg	1 y	1 month a year
Patient 7	Testosterone propionate Methenolone enanthate	200 mg 200 mg	400 mg	1 y	1 month a year
Patient 8	Drostanolone propionate Boldenone Methenolone enanthate Testosterone enanthate	200 mg 500 mg 200 mg 500 mg	1400 mg	4 y	2 months a year
Patient 9	Drostanolone propionate Trenbolone Testosterone enanthate	500 mg 500 mg 500 mg	1500 mg	1 y	4 months a year
Patient 10	Testosterone propionate Drostanolone propionate Nandrolone decanoate	500 mg 300 mg 400 mg	1200 mg	6 y	4 months a year
Patient 11	Testosterone propionate Metenolone	125 mg 50 mg	175 mg	1 y	2 months a year
Patient 12	Testosterone propionate Testosterone enanthate Nandrolone decanoate	500 mg 500 mg 500 mg	1500 mg	3 y	2 months a year

Weekly numbers of training sessions for participants were identified to vary between 4 and 6. All participants using creatine were identified to use 5 g/day dose of creatine. In comparison, the use of creatine among DS users was significantly high (p=0.041). The use rates of multivitamins and herbal extracts were similar in both groups (Table 3). The daily protein powder amounts used by AAS+DS and DS users were similar (44.4±21.3 g/day and 37.8±30 g/day, respectively, p=0.454).

**Table 3:** DS use and habits of participants. DS: Dietary supplement; AAS: Anabolic androgenic steroid. * Pearson Exact Chi-Square Test ** Fisher Exact Chi-Square Test

	Dietary supplement use by AAS users	Dietary supplement use by DS users	P-value
Protein	50%	100%	0.018 *
Creatine	8%	58%	0.041 **
Multivitamin	66%	33%	0.221 *
Herbals extracts	25%	8%	0.584 **
Stimulants	0%	0%	-
Smoking (yes/no)	6/6 (50%/50%)	0/12 (0%/100%)	0.018 **
Alcohol use (yes/no)	3/9 (25%/75%)	3/9 (25%/75%)	1 *

All participants stated they had not used any banned drugs/stimulants during their lives. AAS+DS users drank alcohol at social levels, and DS users were similar. Half AAS+DS users smoked, while DS users were not identified to smoke (Table 3). Cases using AAS+DS were found to have significantly higher levels of aspartate aminotransferase (AST), alanine aminotransferase (ALT), and creatine kinase (CK) compared to DS users (p<0.001 for each). Lactate dehydrogenase (LDH) levels were within the normal interval, though higher for AAS users (p=0.006). The high-density lipoprotein cholesterol level (HDL-C) was within the normal range for AAS users but was lower compared to DS users (p=0.010). Total cholesterol (TC), triglyceride (TG), and low-density lipoprotein cholesterol (LDL-C) were similar in both groups (p=0.688, p=0.102, p=0.191). As expected, the total testosterone level was significantly higher for patients using AAS+DS (p=0.015). Spot urine albumin/creatinine and protein/creatinine ratios were within the normal reference interval, though higher for AAS+DS users (p<0.001, p=0.006, respectively) (Table 4).

**Table 4:** Laboratory values of the participants. Hb: Hemoglobin; BUN: Blood Urea Nitrogen; SCr: Serum Creatinine; AST: Aspartate Aminotransferase; ALT: Alanine Aminotransferase; CK: Creatine Kinase; LDH: Lactate Dehydrogenase; TC: Total Cholesterol; TG: Triglyceride; LDL-C: Low-Density Lipoprotein Cholesterol; HDL-C: High-Density Lipoprotein Cholesterol; TSH: Thyroid Stimulating Hormone; eGFRCr: Creatinine-Based Estimated Glomerular Filtration Rate; eGFRcys/eGFRCr: Glomerular Filtration Rate Based on the Combination of Cystatin C and Creatinine; eGFRcys: Cystatin-Based Estimated Glomerular Filtration Rate. * Independent Sample t-test **Mann-Whitney U test

	AAS+DS	DS	P-value
Hb (13.5-16.9) g/dL	16.47±0.842	16.01±0.99	0.596 *
WBC (4.49-12.68) 103/uL	7.01±1.5	7.1±1.9	0.766 *
Platelet (173-390)103/uL	239±55	249±53	0.429 *
Glucose mg/dl (70-110)	79.92±6.97	82.25±12.7	0.622 *
BUN (8-23)mg/dl	15.2±5.2	15.20±5.4	0.843 *
SCr (0.5-1.2) mg/dl	1.04±0.2	1.03±0.1	0.662 *
Uric acid (2.4-5.7)mg/dl	5.51±1.0	5.47±1.3	0.411 *
AST (0-31)U/L	41.92±30	26.83±10.4	<0.001
ALT (0-33)U/L	66.58±92.8	30.08±15.04	<0.001
CK (34-170) U/L	643.67±601	394.25±325	<0.001
LDH (135-214) U/L	212.25±49	201.75±40	0.006 *
TC (118-199) mg/dl	170.92±35.8	178.50±34.6	0.688 *
TG (60-149)mg/dl	95.92±52.14	106.75±43	0.102 *
HDL-C (40-63)mg7dl	40.00±12.24	52.50±14.79	0.010 *
LDL-C (88-159)mg/dl	121.17±36.66	115.92±31.0	0.191 *
TSH uIU/ml (0.51-4.3)	2.04±0.82	1.96±0.42	0.205 *
Testosterone ng/dl (249-836)	819.50±491.03	470.90 ± 121.7	0.015 **
Urine protein/creatinine ratio(<150mg/day)	69.79±33.44	57.98±15.31	0.006 **
Urine albumin/creatinine ratio (<30 mg/day)	7.47±12.4	4.73±5.64	<0.001
Serum human cystatin C (mg/L) (0.28-2.2)	0.91±0.13	0.88±0.16	0.263 *
eGFRCr	119.67±24.12	122.08±18.03	0.426 *
eGFRcys/eGFRCr	121.83±20.62	126.33±21.163	0.036 *
eGFRcys	120.67±19.48	127.33±27.526	0.039 **

Serum cystatin C levels were similar in groups using AAS+DS and DS (0.91±0.13 mg/L, 0.88±0.16 mg/L, respectively, p=0.263). The eGFRcr was similar in the groups using AAS+DS and DS (119.67±24.12 ml/min, 122.08±18.03 ml/min, p=0.426). The eGFRcys and the eGFRcys/cr were found to be significantly lower in the group using AAS+DS compared to the group using DS (eGFRcys, 120.67 ± 19.48 ml/min, 122.08 ± 18.03 ml/min, p = 0.039, eGFRcys/cr 121.83 ± 20.62 ml/min, 126.33 ± 21.163 ml/min, p = 0.036, respectively). The longitudinal length of both right and left kidneys was similar for AAS+DS users and DS users (p=0.128, p=0.617, respectively) (Table 4). The side effects and rates related to AAS use among AAS+DS users are summarized in Table 5.

**Table 5:** Side effect rates of 12 bodybuilders using AAS+DS. AAS: Anabolic androgenic steroid; DS: Dietary supplement.

Side Effects	%
Nausea	8.3
Headaches	16.7
Anxiety	41.7
Depression	16.7
Altered libido	41.7
Site reaction	16.7
Gynecomastia	25
Acne	50
Testicular atrophy	33.3

## Discussion

In our study, bodybuilders using AAS+DS and DS had similar creatinine-based eGFR values. In contrast, AAS+DS users had lower eGFR values based on cystatin C and the combination of cystatin C and creatinine. Those using AAS+DS had higher total proteinuria and albuminuria, though values were within the normal reference interval. Though several adverse health effects secondary to taking anabolic steroids are well known, renal injury, especially linked to FSGS, is less recognized. For the first time, Herlitz LC et al. reported 10 patients with a diagnosis of FSGS due to a combination of post-adaptive glomerular changes developing as a result of direct nephrotoxic effects potentially due to increased fat-free body mass and anabolic steroid use in bodybuilders. They proposed that this complication was not sufficiently recognized due to the expected increase in serum creatinine due to increased body mass in bodybuilders [6]. Almukhtar SE et al. performed renal biopsies on four bodybuilders with kidney dysfunction taking anabolic steroids, commercial protein, and creatine products. They reported acute tubular necrosis, with chronic injury findings like >30% interstitial fibrosis and tubular atrophy in two biopsies. These findings were proposed to emphasize the risk of acute and potentially chronic renal injury among young men using anabolic steroids and excessive amounts of nutritional supplements [16]. During a six-year period, El-Reshaid W et al. performed a renal biopsy on 22 adult men with a high-protein diet and AAS or growth hormone (GH) use. In eight cases, FSGS was identified, four had nephroangiosclerosis, three had chronic interstitial nephritis, two had acute interstitial nephritis, two had chronic interstitial nephritic nephrocalcinosis, and one patient each had membranous glomerulopathy, crescentic glomerulopathy, and sclerosing glomerulonephritis. It has been reported that patients with FSGS have a longer exposure time, late presentation, and a worse prognosis. In comparison, those with interstitial disease have a shorter exposure time and earlier presentation, and renal function improves or stabilizes after discontinuation of applications [17]. The normal value for GFR is influenced by age, sex, and body size, and there can be significant differences even among healthy individuals, with values around 130 mL/min/1.73 m² for men and 120 mL/min/1.73 m² for women, respectively [18]. The normal albumin excretion rate is less than 10 mg per day [19]. Increased albuminuria is the earliest biomarker for underlying microvascular disease in the kidneys, and an increase in albuminuria over time is known to be associated with a loss of GFR [20].

FSGS develops as an adaptive process in obese patients with larger body mass causing glomerular hyperfiltration, leading to mechanical difficulty and scarring over time. The increasing BMI of bodybuilders is thought to contribute to secondary FSGS development [6, 21]. In our study, participant BMI was 25-30 kg/m2, so participants were not obese, and both groups had similar BMI. Though the potential effects of AAS on renal functions in humans are not well-defined, several studies showed that androgens may have direct toxic effects on glomerular cells and led to the consideration that this may cause mesangial matrix accumulation and podocyte consumption, independent of structural-functional adaptations. In an adriamycin nephropathy model, male rats developed more glomerulosclerosis than female rats. Treatment of female rats with ovariectomy or exogenous testosterone administration caused an increase in albuminuria and acceleration of mesangial sclerosis [22]. Androgen receptors were identified in micro-dissected murine glomeruli and cultured mesangial cells. Exogenous testosterone administration increased androgen receptor expression in addition to profibrotic cytokine TGF-1 mRNA levels [23]. Additionally, in transgenic models, excessive expression of TGF-1 was shown to support FSGS by providing strong proapoptotic stimulation of podocytes [24]. Though participants in our study appeared to have normal renal functions and albumin excretion rates in urine, AAS+DS users had significantly lower cystatin C-based GFR values than DS users and significantly higher amounts of albumin excretion. These findings bring to mind a correlation between early renal injury linked to direct AAS use and the need for more research on the topic.

Protein supplementation causes an increase in renal perfusion and GFR as an appropriate adaptive response to the increase in nitrous waste, a product of protein metabolism. This situation is considered to accelerate chronic hyperfiltration and progression to glomerulosclerosis [25]. Recent data show that moderate protein supplementation of 60 g/day for one month does not cause adverse effects on renal function in overweight and obese individuals with normal renal function. Recent data show moderate degrees of protein supplementation, like 60 g/day for one-month duration, may cause unwanted effects on renal function in overweight and obese individuals with normal renal function [26]. Additionally, there is a need to research the impact of higher doses and long-duration protein supplementation. However, the effect of higher-dose and long-term protein supplementation on kidney function needs to be investigated. In our study, while the rate of protein use was naturally higher in the DS group, the amount of protein consumed by both groups was found to be similar since the daily protein intake was higher in the patients using protein in the ASS+DS group. The similar consumption amounts for protein in both groups lead to the conclusion that the differences in albuminuria and cystatin-based estimated GFR may be linked to AAS use. Bodybuilders frequently use creatine, and this elevates plasma creatinine levels and may show eGFR as mistakenly low [27]. However, a new meta-analysis showed that creatine supplementation did not significantly change creatinine levels and emphasized the need for more homogeneous and high-quality studies [28]. In our study, the creatine use rates were significantly high in the group using DS, and this situation may have caused high measurement of serum creatinine levels in patients and low calculation of GFRCr values. This may explain the similar measurement of GFRCr values in both groups.

Human studies have not directly addressed the potential role of androgens in podocyte damage or hemodynamic factors as predisposing to the development of FSGS. Given the widespread use of AASs among athletes, it is likely that additional genetic and environmental factors are required to establish clinically significant kidney disease. Deshmukh N et al. reported that with chronic and /or excessive use of AAS, individuals with a deletion polymorphism in the UGT2B17 gene (del/del) may be at increased risk of developing kidney disorders due to the increase in BMI and possible direct toxic effects of steroids on the kidneys. They hypothesized that insufficient elimination of biologically active steroids would cause elevated serum levels and excessive increase in muscle mass, and that the increase in BMI could cause sustained high glomerular pressure and kidney injuries. They reported that further research on AAS metabolism in the presence of UGT2B17 gene deletion is required [29]. In athletes using AAS and other performance-enhancing drugs and supplements, proteinuria and mild kidney failure are generally asymptomatic, and kidney injury will probably be inadequately diagnosed if not regularly screened. The need for closer monitoring of kidney functions should be considered in the presence of gene deletions that may cause possible slowing of AAS metabolism. Among additional side effects associated with AAS, changes in blood lipid levels (increase in LDL and reduction in HDL) and a variety of hepatotoxic side effects are reported [30]. In our study, HDL level was low among AAS users with high liver enzyme levels. Our study is the first pilot study to use creatinine in conjunction with cystatin C to assess kidney functions in bodybuilders. The participants were carefully chosen, and patients with hypertension, diabetes mellitus, or a single kidney (confirmed with renal ultrasound) that could cause hyperfiltration injury were not included in the study. Additionally, our study has some limitations. The first of these is the low number of participants, which was due to the closure of gyms linked to the COVID-19 pandemic and the refusal of some bodybuilders to participate in the study, as they wanted to keep their drug use confidential. Another limitation is that participants used AAS and diet supplements periodically according to competition or active definition periods, whereas our study was conducted randomly, independent of these periods. However, we believe that our findings are still valuable in terms of assessing the renal impacts of long-term drug use.

## Conclusions

Cystatin-based GFR values were found to be significantly lower in AAS+DS users and significantly higher in urinary albumin and protein excretion than in DS users. Although these findings suggest a relationship between early kidney damage directly related to the use of AAS, the subject needs to be investigated further.
